# Expression of Tumor Suppressor *FHIT* Is Regulated by the *LINC00173*-SNAIL Axis in Human Lung Adenocarcinoma

**DOI:** 10.3390/ijms242317011

**Published:** 2023-11-30

**Authors:** Takahito Suzuki, Satoshi Sakai, Kosuke Ota, Mika Yoshida, Chiharu Uchida, Hiroyuki Niida, Takafumi Suda, Masatoshi Kitagawa, Tatsuya Ohhata

**Affiliations:** 1 Department of Molecular Biology, Hamamatsu University School of Medicine, Hamamatsu 431-3192, Japan; 2 Second Division, Department of Internal Medicine, Hamamatsu University School of Medicine, Hamamatsu 431-3192, Japan; 3 Advanced Research Facilities & Services, Preeminent Medical Photonics Education & Research Center, Hamamatsu University School of Medicine, Hamamatsu 431-3192, Japan

**Keywords:** lncRNA, *LINC00173*, SNAIL, FHIT, lung adenocarcinoma, tumor suppressor gene

## Abstract

Long non-coding RNAs (lncRNAs) play a critical role in a variety of human diseases such as cancer. Here, to elucidate a novel function of a lncRNA called *LINC00173*, we investigated its binding partner, target gene, and its regulatory mechanism in lung adenocarcinoma, including the A549 cell line and patients. In the A549 cell line, RNA immunoprecipitation (RIP) assays revealed that *LINC00173* efficiently binds to SNAIL. RNA-seq and RT-qPCR analyses revealed that the expression of *FHIT* was decreased upon *LINC00173* depletion, indicating that *FHIT* is a target gene of *LINC00173*. Overexpression of SNAIL suppressed and depletion of SNAIL increased the expression of FHIT, indicating that SNAIL negatively regulates FHIT. The downregulation of *FHIT* expression upon *LINC00173* depletion was restored by additional SNAIL depletion, revealing a *LINC00173*-SNAIL-FHIT axis for FHIT regulation. Data from 501 patients with lung adenocarcinoma also support the existence of a *LINC00173*-SNAIL-FHIT axis, as *FHIT* expression correlated positively with *LINC00173* (*p* = 1.75 × 10^−6^) and negatively with *SNAIL* (*p* = 7.00 × 10^−5^). Taken together, we propose that *LINC00173* positively regulates *FHIT* gene expression by binding to SNAIL and inhibiting its function in human lung adenocarcinoma. Thus, this study sheds light on the *LINC00173*-SNAIL-FHIT axis, which may be a key mechanism for carcinogenesis and progression in human lung adenocarcinoma.

## 1. Introduction

RNA molecules that have little or no coding ability and are generally more than 200 nucleotides in length are so called long non-coding RNAs (lncRNAs) [1]. The definition of lncRNAs is based solely on their length and coding capacity; however, they are involved in a variety of biological processes and have a wide range of molecular functions. LncRNAs are not only associated with physiological processes, such as cell proliferation, differentiation, and apoptosis, but also with pathological processes, such as myopathy, senescence, and tumorigenesis [2,3,4]. LncRNAs upregulate translation of their target mRNAs by binding to microRNAs (miRNAs) of the target genes and acting as so-called molecular sponges [5]. Another important function of lncRNAs is to selectively bind to target proteins, including transcription factors, signal transducers, and epigenetic modifiers, to modulate their activity, stability, complex formation, and cellular localization [4,6]. Long intergenic non-protein coding RNA 173 (*LINC00173*) is a long intergenic non-coding RNA (lincRNA), a type of lncRNA that does not overlap with protein-coding genes. *LINC00173* is associated with various types of human cancer in a cell context-dependent manner. It is involved in promoting tumorigenesis and tumor progression in squamous cell carcinoma, small cell lung cancer, hepatocellular carcinoma, and colorectal cancer [7]. In contrast, it also suppresses tumorigenesis and tumor progression in lung adenocarcinoma [8], cervical cancer [9], and acute myeloid leukemia [10]. This cell context-dependent action of *LINC00173* can be attributed to its varied molecular functions; it acts as a molecular sponge of miRNAs to stabilize the mRNAs and enhance the expression of target genes [7], and it selectively binds to target proteins, such as DNMT1 [10] and HNRNPA2B1 [11], to modulate their downstream pathways. This variety of *LINC00173* molecular actions indicates that *LINC00173* may have as yet undiscovered functions in other important biological processes.

The SNAIL family encodes zinc finger transcription factors and includes SNAIL (also referred to SNAI1/SNAIL1), SLUG (SNAI2/SNAIL2), and SMUC (SNAI3/SNAIL3). The SNAIL family is involved in physiological events such as morphogenesis via mesoderm specification, neural crest specification, and epithelial-mesenchymal transition (EMT) [12,13,14]. Additionally, the SNAIL family is also associated with cell stemness [15], chemoresistance [15], anti-apoptosis [16], angiogenesis [17], and immune checkpoint signaling [18], all of which affect cancer progression and recurrence. SNAIL contains a SNAG domain in the N-terminal region, a serine-rich domain and a nuclear export sequence in the central region, and four C2H2 zinc finger motifs in the C-terminal region [19]. The SNAG domain is critical for the association with transcriptional co-repressors, including histone deacetylase 1/2 (HDAC1/2) [20], polycomb repressive complex 2 (PRC2) [21], lysine demethylase 1 (LSD1, also referred to KDM1A) [22], and suppressor of variegation 3–9 homolog 1 (SUV39H1) [23]. These co-repressors promote target gene silencing by introducing repressive epigenetic modifications [13,24]. The serine-rich domain is critical for regulating the stability of SNAIL protein through ubiquitin-mediated degradation via its association with E3 ubiquitin ligases, including SCF-FBXL14 [25] and SCF-β-TrCP [26], and for functional regulation of SNAIL protein through various post-translational modifications, including phosphorylation [12,13,14,27]. The nuclear export sequence motif is critical for translocation of the SNAIL protein from the cytoplasm to the nucleus by phosphorylation of an adjacent serine-rich domain that makes the nuclear export sequence accessible to the CRM1 transporter [28]. The zinc finger motif facilitates SNAIL protein recruitment to target genes by binding to the E-box in the regulatory region of the target genes. For example, when SNAIL represses the *CDH1* gene during EMT progression, the zinc finger motif binds to the E-box in the *CDH1* promoter and the SNAG domain recruits HDAC1/2-Sin3A, which introduces a repressive histone modification, histone deacetylation [14,20,29]. The function and regulation of SNAIL is also controlled by several lncRNAs that act as molecular sponges of miRNAs to stabilize the SNAIL mRNA and enhance its translation [30,31]. LncRNAs may also regulate SNAIL function by binding to the SNAIL protein; however, this action has not been well characterized.

The Fragile histidine triad gene (*FHIT*) maps to a chromosome 3 region called FRA3B, which is one of the most fragile of the common fragile sites (CFSs) in human cancers [32,33,34]. CFSs are genomic loci characterized by AT-rich sequences, complex replication, and transcriptional repression, and are prone to breakage and gap formation in metaphase chromosomes [35]. Because of the characteristics of CFSs, it was previously thought that deletions at the FRA3B locus found in many cancers were simply passenger events rather than a cause of loss of *FHIT* function [32,33,34]. However, much experimental evidence supports *FHIT* as a tumor suppressor gene, including human lung [36,37,38], breast [39], cervical [37], esophageal [40], gastric [36], pancreatic [41], and renal cancer [42]. For example, introduction of the *FHIT* gene into several esophageal cancer cell lines lacking *FHIT* induced Caspase-dependent apoptosis and cell cycle arrest [40]. *Fhit* knockout mice are prone to tumor development, and reintroduction of the wild-type *Fhit* gene by adenoviral transfection into *Fhit* knockout mice facilitated the recovery of tumor incidence [43]. Furthermore, introduction of *FHIT* into pancreatic cancer cells, from which most *FHIT* had been deleted, induced apoptosis, delayed tumor growth, and prolonged survival in a mouse model [41]. *FHIT* also negatively regulates EMT through negatively regulating an EGFR/Src/ERK/Slug signaling axis in human bronchial cells [44] and by positively regulating miR-30c expression, which suppresses EMT and metastasis by directly targeting metastasis genes Metadherin (*MTDH*), High-mobility group AT-hook2 (*HMGA2*), and the mesenchymal markers Vimentin (*VIM*) and Fibronectin (*FN1*) in human lung cancer [45]. However, the regulatory mechanism of *FHIT* expression, especially by lncRNAs, is poorly understood.

We explored novel functions of *LINC00173* by performing RNA immunoprecipitation (RIP) assays and found that *LINC00173* efficiently binds to SNAIL. RNA-sequencing (RNA-seq) and reverse transcription quantitative polymerase chain reaction (RT-qPCR) analyses revealed that the expression of *FHIT* was decreased upon *LINC00173* depletion, indicating that *FHIT* is the target gene of *LINC00173*. The *FHIT* gene promoter contains several SNAIL binding sequences, called E-boxes. Overexpression of SNAIL suppressed *FHIT* expression and depletion of SNAIL increased *FHIT* expression, indicating that SNAIL negatively regulates *FHIT* expression. Data from lung adenocarcinoma patients showed that *FHIT* expression is positively correlated with *LINC00173* and negatively correlated with SNAIL. Taking these findings together, we propose that *LINC00173* increases *FHIT* gene expression by binding to SNAIL and inhibiting its function.

## 2. Results

### 2.1. lncRNA LINC00173 Binds to Transcription Factor SNAIL in A549 Cells

**Figure 1:**
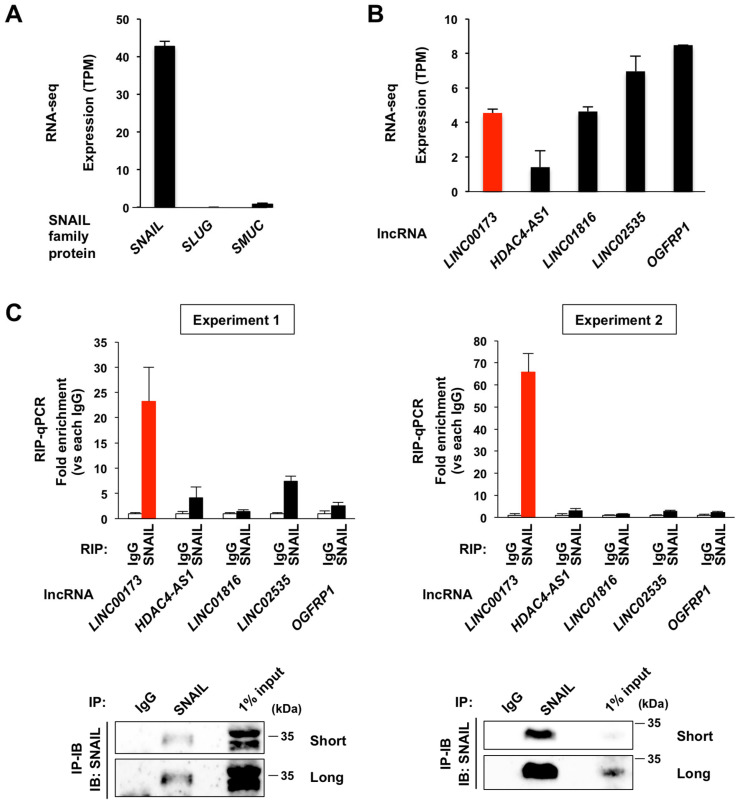
*LINC00173* binds to SNAIL in A549 cells. (**A**) The gene expression level of SNAIL family protein in A549 cells, obtained by RNA-seq. (**B**) The gene expression level of *LINC00173* and of four randomly selected lncRNAs in A549 cells, obtained by RNA-seq. (**C**) RIP assay results using anti-SNAIL antibody with the lncRNAs are shown in the upper panel and confirmation of immunoprecipitation by immunoblotting (IB) of immunoprecipitation (IP) samples is shown in the lower panel. The result is confirmed in two independent biological replicates as shown in the first experiment (**left**) and the second experiment (**right**). Short and Long indicate short and long exposure times, respectively. See also Figure S2 for the uncropped images.

*LINC00173* is associated with various types of human cancer in a cell context-dependent manner [7,9,10,46]. Among them, we focused on the non-small cell lung cancer, lung adenocarcinoma, in which *LINC00173* acts as a suppressor of tumorigenesis and tumor progression [8,46]. We selected the A549 cell line derived from human lung adenocarcinoma for the analysis and obtained its expression profile under our culture conditions by RNA-seq. To discover novel functions of *LINC00173*, we first focused on the SNAIL family of zinc finger transcription factors, involved in cancer progression and recurrence in various human cancers, including lung cancer [47,48], as a candidate interacting partner of *LINC00173*. Among the SNAIL family protein, we chose *SNAIL* for RIP-qPCR analysis because *SLUG* and *SMUC*, the other member of SNAIL family protein, were barely expressed in the A549 cells (Figure 1A). From our lncRNA primer set collection, we selected four lncRNAs for RIP-qPCR analysis, which had lower (*HDAC4-AS1*), comparable (*LINC01816*), and higher levels of expression (*LINC02535* and *OGFRP1*) compared with the level of *LINC00173* expression based on the expression profile of A549 cell line (Figure 1B), together with *LINC00173.* Five *LINC00173* variants are registered in the Ensembl database (GRCh38.p13) (Figure S1A). To analyze the expression levels of each *LINC00173* variant, we used our RNA-seq-derived expression profile of A549 cells and found that *LINC00173*-205 had the highest expression level, followed by *LINC00173*-203 (Figure S1B). Only *LINC00173*-203, and not *LINC00173*-205, has been previously functionally analyzed [8,49,50,51] (note that variant *LINC00173*-203 registered in the Ensembl database is identical to variant NR_027345.1, also called *LINC00173*-TSV1, registered in the NCBI database). Based on these expression profiles and previous reports, we decided to focus on *LINC00173*-203 (we hereafter refer to *LINC00173*-203 as *LINC00173*). We designed a primer set that specifically identifies this variant (Figure S1C) and performed RIP-qPCR analysis. The results showed that *LINC00173* was highly bound to SNAIL in A549 cells in two independent experiments (Figure 1C and Figure S2). The amount of *LINC00173* RNA bound to SNAIL was more pronounced than for *LINC02535* and *OGFRP1* (Figure 1C and Figure S2), which are both expressed at higher levels than *LINC00173* in A549 cells (Figure 1B), indicating stringent binding of SNAIL to *LINC00173* compared with other lncRNAs. From these data, we concluded that the transcription factor, SNAIL, is a binding partner of *LINC00173* in A549 cells.

### 2.2. Identification of FHIT as a Target Gene for LINC00173

**Figure 2:**
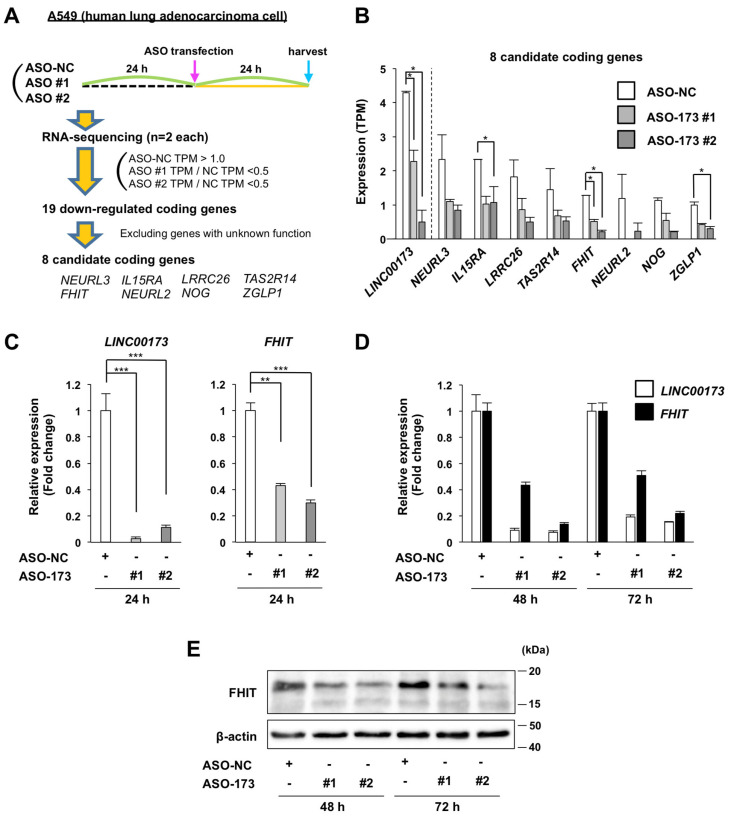
Identification of *FHIT* as a target gene of *LINC00173*. (**A**) Workflow for screening target genes of *LINC00173*. ASO #1: ASO-*LINC00173*_#1; ASO #2: ASO-*LINC00173*_#2. (**B**) Downregulation of eight candidate *LINC00173* target genes upon *LINC00173* depletion, confirmed by RNA-seq. (**C–D**) Downregulation of *FHIT* mRNA, 24 h (**C**), 48 h, and 72 h (**D**) after transfection of ASO-173, confirmed by RT-qPCR (normalized to *GAPDH*, relative expression to ASO-NC transfection at each time point is shown). (**E**) Downregulation of FHIT protein, 48 h and 72 h after transfection of ASO-173, confirmed by western blotting. A representative image is shown from three independent biological replicates. (**B**–**D**) Mean ± SD relative to the mean of each ASO-NC. *: *p* < 0.05, **: *p* < 0.01, and ***: *p* < 0.001, relative to each ASO-NC transfection, Student’s t-test. (**B**): two biological replicates, (**C**): three biological replicates, and (**D**): one biological experiment with three experimental replicates are shown. ASO-NC: ASO negative control, ASO-173: ASO-*LINC00173*. See also Figure S5 for the uncropped images.

To identify target genes of *LINC00173* involved in human cancer, we performed RNA-seq analysis on RNA samples prepared from A549 cells transfected with two independent antisense oligonucleotides (ASOs) for *LINC00173* (ASO-173 #1 and #2) and a negative control ASO (ASO-NC) (Figure 2A). Adequate efficiency of *LINC00173* knockdown was confirmed by RNA-seq (Figure 2B). To identify *LINC00173* target genes, we first selected coding genes with more than 1.0 transcripts per million (TPM) at baseline (i.e., from the samples transfected with ASO-NC). Among these, we then selected genes with a less than 0.5-fold decrease in TPM following *LINC00173* knockdown by both independent ASOs and obtained 19 down-regulated coding genes. We excluded 11 of these genes whose biological function was not annotated. The remaining eight coding genes, *NEURL3*, *IL15RA*, *LRRC26*, *TAS2R14*, *FHIT*, *NEURL2*, *NOG,* and *ZGLP1*, were selected as candidate target genes for *LINC00173* and further analyzed (Figure 2A and Table S1). Statistical analysis revealed that only *FHIT* was significantly downregulated in both ASO-173 #1 and #2 *LINC00173* knockdown samples compared with the control (ASO-NC) sample (Figure 2B and Table S1). These eight candidate genes were subjected to RT-qPCR analysis to confirm reproducibility, and only *FHIT* and *NEURL3* showed significant down-regulation upon *LINC00173* depletion in both of the independent knockdown samples (Figure 2C and Figure S3). *FHIT* is a known tumor suppressor gene and is downregulated in various human cancers [33]. In contrast, the function of *NEURL3* is not well understood and its association with cancer has not been reported. Therefore, we decided to focus on *FHIT* as a *LINC00173* target gene. The down-regulation of *FHIT* expression upon *LINC00173* depletion was observed not only in A549 cells but also in non-small cell lung cancer cell line, H1299, and in breast cancer cell line, MDA-MB-231 (Figure 2C and Figure S4). This indicates that the repression mechanism of *LINC00173* on *FHIT* is conserved between lung and breast cancer. The reduction in *FHIT* mRNA levels by *LINC00173* depletion was sustained for up to 48 and 72 h after *LINC00173* knockdown (Figure 2D). The amounts of *FHIT* protein were correspondingly reduced by *LINC00173* depletion at these time points (Figure 2E and Figure S5). These data indicate that *LINC00173* positively regulates *FHIT* expression and that *FHIT* is a bona fide target gene of *LINC00173*.

### 2.3. Regulation of FHIT Expression by the LINC00173-SNAIL Axis

**Figure 3:**
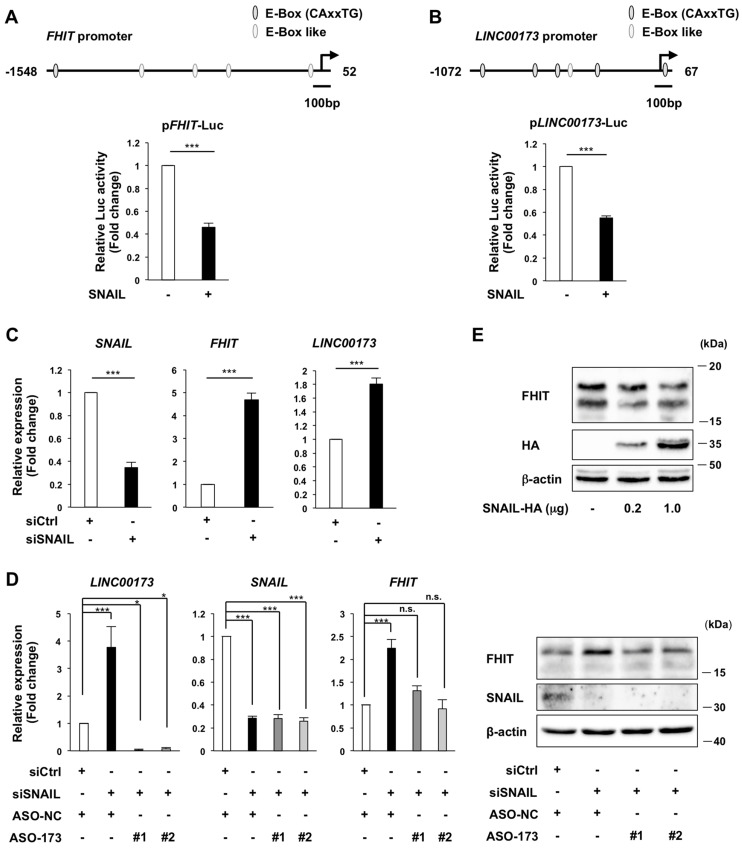
Regulation of *FHIT* expression by the *LINC00173*-SNAIL axis. (**A,B**) *FHIT* (**A**) and *LINC00173* (**B**) promoter activity, upon overexpression of SNAIL, measured by luciferase assays. Schematic representation of *FHIT* (**A**) and *LINC00173* (**B**) promoters with E-box and E-Box-like motifs are shown at the top. (**C**) *SNAIL*, *FHIT*, and *LINC00173* RNA levels upon SNAIL depletion by knockdown measured by RT-qPCR. (**D**) *SNAIL*, *FHIT*, and *LINC00173* RNA levels measured by RT-qPCR (left), FHIT and SNAIL protein levels measured by western blotting (right), upon SNAIL depletion by knockdown, combined with *LINC00173* depletion by ASO. (**E**) FHIT protein levels upon overexpression of SNAIL measured by western blotting. (**A**–**D**) Mean ± SD relative to the mean of each control transfection, normalized to β-gal (**A,B**) and *GAPDH* (**C,D**). n.s: not significant, *: *p* < 0.05, and ***: *p* < 0.001, relative to each control transfection, Student’s *t*-test (**A–C**) and Bonferroni correction (**D**). (**A,B,D**): four biological replicates and (**C**): three biological replicates are shown. (**D,E**) A representative image is shown from three independent experiments. See also Figure S6 for the uncropped images.

*LINC00173* binds to SNAIL and positively regulates *FHIT* expression; therefore, we next examined the involvement of SNAIL in the positive regulation of *FHIT* by *LINC00173*. We analyzed the promoter region of *FHIT* and identified E-box and E-box-like motifs, the sequences to which SNAIL binds, in the *FHIT* promoter. We then examined whether SNAIL regulates *FHIT* expression using luciferase assays. We found that the transcriptional activity of the *FHIT* promoter was reduced by overexpression of SNAIL (Figure 3A). Intriguingly, E-box and E-box-like motifs were also identified in the promoter region of *LINC00173* (Figure 3B). We therefore performed similar luciferase assays on the *LINC00173* promoter and found that the transcriptional activity of the *LINC00173* promoter decreased upon SNAIL overexpression (Figure 3B). Furthermore, significant increases in *FHIT* and *LINC00173* expression were observed upon SNAIL depletion in A549 cells (Figure 3C). This indicates that not only *FHIT* but also *LINC00173* is negatively regulated by SNAIL. We also found that depletion of SNAIL resulted in increased levels of FHIT protein (Figure 3D) and, conversely, that overexpression of SNAIL decreased the level of FHIT protein (Figure 3E). This indicates that the reduction in *FHIT* transcription by SNAIL also decreases the level of FHIT protein, which is responsible for its physiological functions.

*FHIT* expression was obviously reduced upon *LINC00173* depletion (Figure 2C,D). To confirm whether SNAIL, which functions to suppress *FHIT* expression, is involved in the suppression of *FHIT* expression upon *LINC00173* depletion, we additionally knocked down SNAIL under the conditions of *LINC00173* depletion and tested whether the suppression of *FHIT* expression upon *LINC00173* depletion was restored by the additional knockdown of SNAIL. We found that *FHIT* expression, which was obviously decreased upon *LINC00173* depletion (Figure 2C,D), was restored to pre-*LINC00173* depletion levels by additional knockdown of SNAIL (Figure 3D). This indicates that SNAIL is involved in repressing *FHIT* expression upon *LINC00173* depletion. We therefore propose that *LINC00173* positively regulates *FHIT* expression by inhibiting SNAIL function.

### 2.4. Impact of the LINC00173-SNAIL-FHIT Axis on Human Lung Adenocarcinoma

**Figure 4:**
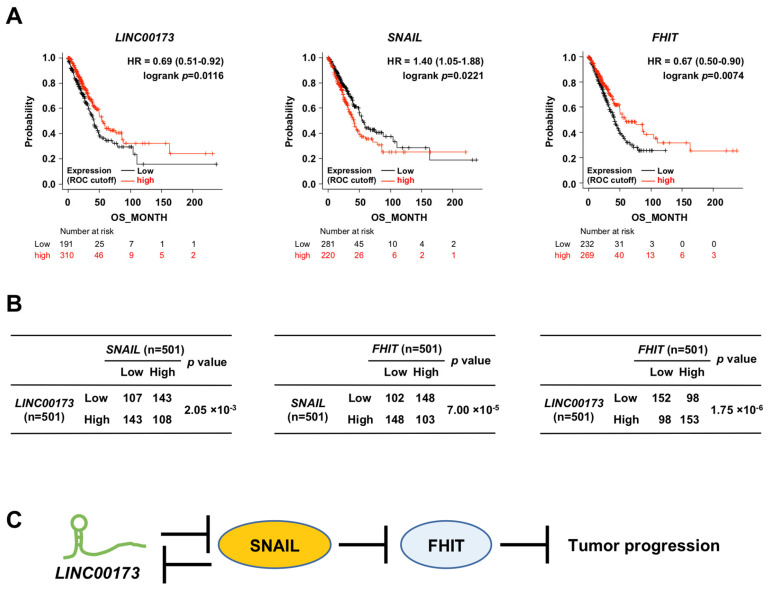
Impact of the *LINC00173*-SNAIL-FHIT axis on human lung adenocarcinoma. (**A**) Prognostic analysis of patients with lung adenocarcinoma expressing *LINC00173*, *SNAIL*, and *FHIT* using Kaplan-Meier analysis. (**B**) Correlation analysis between *LINC00173* and *SNAIL*, *SNAIL* and *FHIT*, and *LINC00173* and *FHIT* in human lung adenocarcinoma using the χ^2^ test. (**C**) A model of the *LINC00173*-SNAIL-FHIT axis in human lung adenocarcinoma. HR: hazard ratio, ROC: receiver operating characteristic, OS: overall survival.

We next examined whether the *LINC00173*-SNAIL-FHIT axis identified in the lung adenocarcinoma cell line A549 is also observed in cancer patients. A lung adenocarcinoma dataset was selected from cBioportal (lung adenocarcinoma, TCGA, PanCancer Atlas, *n* = 566). From this dataset we selected samples with *LINC00173*, *SNAIL*, and *FHIT* expression data and overall survival status of patients (living or deceased). A total of 501 samples met these conditions. Using this dataset, we performed Kaplan–Meier analysis and found that the prognosis of the patient group with low *LINC00173* expression, high SNAIL expression, and low *FHIT* expression was significantly poor (*p* = 0.0116, 0.0221, and 0.0074, respectively, Figure 4A). This is consistent with previous reports showing that *LINC00173* and *FHIT* act as tumor suppressors in lung adenocarcinoma [8,46,52]. This dataset was then used for correlation analysis between the expression of these genes. The results showed a significant negative correlation between *LINC00173* and SNAIL, a significant negative correlation between SNAIL and *FHIT*, and a significant positive correlation between *LINC00173* and *FHIT* (*p* = 2.05 × 10^−3^, 7.00 × 10^−5^, and 1.75 × 10^−6^, respectively, Figure 4B). This indicates that the *LINC00173*-SNAIL-FHIT axis also exists in patients with lung adenocarcinoma, where *LINC00173* acts as a tumor suppressor.

We then performed a similar analysis using a breast cancer dataset (Breast invasive carcinoma, TCGA PanCancer Atlas, *n* = 1082), the tissue of origin of the MDA-MB-231 cell line in which we observed reduced *FHIT* expression upon depletion of *LINC00173* (Figure S4B). Kaplan–Meier analysis showed that only the group of patients with low *FHIT* expression, but not low *LINC00173* expression and high *SNAIL* expression, had a significantly poor prognosis (*p* = 0.00394, Figure S7A). In contrast, correlation analysis showed a significant negative correlation between *LINC00173* and *SNAIL*, a significant negative correlation between *SNAIL* and *FHIT*, and a significant positive correlation between *LINC00173* and *FHIT* (*p* = 9.77 × 10^−12^, 1.70 × 10^−7^, and 2.03 × 10^−23^, respectively, Figure S7B), comparable to the results from lung adenocarcinoma (Figure 4B). This indicates that there may be a regulatory mechanism involving a *LINC00173*-SNAIL-FHIT axis in breast cancer, but that it may not convey a large enough effect to affect the breast cancer prognosis. Subsequently, we performed a similar analysis using a lung squamous cell carcinoma dataset (Lung squamous cell carcinoma, TCGA PanCancer Atlas, *n* = 478), which is another category of non-small cell lung cancer. In contrast to lung adenocarcinoma, the lung squamous cell carcinoma dataset results did not support involvement of the *LINC00173*-SNAIL-FHIT axis in cancer prognosis; Kaplan–Meier analysis showed that only the group of patients with low *SNAIL* expression had a significantly poor prognosis (*p* = 0.00104, Figure S7C), and correlation analysis showed that although a significant negative correlation was observed between *LINC00173* and *SNAIL* (*p* = 0.00187, Figure S7D), a positive correlation was observed between *LINC00173* and *FHIT* (*p* = 0.0104, Figure S7D), and no significant correlation was observed between *SNAIL* and *FHIT* (*p* = 0.360, Figure S7D). 

Finally, we performed a similar analysis using an acute myeloid leukemia dataset (Acute myeloid leukemia, TCGA PanCancer Atlas, *n* = 149) and a cervical squamous cell carcinoma dataset (Cervical squamous cell carcinoma, TCGA PanCancer Atlas, *n* = 294) in which *LINC00173* suppresses tumorigenesis and tumor progression [9,10]. Using the acute myeloid leukemia dataset, we performed Kaplan–Meier analysis and correlation analysis (Figure S8A,B); however, no significant difference is observed in these analyses, except for the correlation analysis between *LINC00173* and *SNAIL* (*p* = 0.0014, Figure S8B), which was positive but not negative correlation as observed in lung adenocarcinoma (Figure 4C). Using the cervical squamous cell carcinoma dataset, we performed Kaplan–Meier analysis and found that the prognosis of the patient group with low *LINC00173* expression was significantly poor (*p* = 0.018, Figure S8C), which is consistent with a previous report showing that *LINC00173* acts as a tumor suppressor [10]. However, the Kaplan–Meier analysis of *SNAIL* and *FHIT*, as well as all of the correlation analysis showed no significant difference between the comparison (Figure S8C,D). This indicates that the *LINC00173*-SNAIL-FHIT axis and its impact on prognosis in cancer patients is cancer cell context-dependent. Taking these findings together, we conclude that the *LINC00173*-SNAIL-FHIT axis identified in the A549 cell line is also present in patients with lung adenocarcinoma.

## 3. Discussion

In this study, we propose a model in which *LINC00173* positively regulates *FHIT* expression by repressing SNAIL function in human lung adenocarcinoma (Figure 4C). In this *LINC00173*-SNAIL-FHIT axis, *FHIT* acts as a tumor suppressor to inhibit carcinogenesis and tumor progression in lung cancer [32,33,34]. Our results using human lung adenocarcinoma samples are consistent with this because a significant positive correlation was observed between lower expression of *FHIT* and worse prognosis. This raises the question of what triggers the suppression of *FHIT* expression in human lung adenocarcinoma. Based on our new findings, we first propose a pathway whereby loss of *LINC00173* restores SNAIL function and suppression of *FHIT* expression (i.e., a *LINC00173*-SNAIL-FHIT axis). This is consistent with the significant positive correlation between lower expression of *LINC00173* and worse prognosis of lung adenocarcinoma. We found that *LINC00173* transcription is negatively regulated by SNAIL (Figure 4C); therefore, we also propose an alternative pathway in which increased expression of SNAIL triggers the repression of *LINC00173* expression, thereby further improving SNAIL function and consequently decreasing *FHIT* expression. Indeed, we observed a significant positive correlation between higher SNAIL expression and worse prognosis in human lung adenocarcinoma (please note that in addition to suppressing *FHIT* expression, SNAIL has many other known functions that contribute to worsen cancer prognosis [13,14,48], and these mechanisms may also be involved in the worse lung adenocarcinoma prognosis). Although the detection of *FHIT* may be sufficient for prognosis, the expression status and genetic defects of *LINC00173* in pre-cancerous or actual lung adenocarcinoma may also be a potential biomarker for carcinogenesis and progression of lung adenocarcinoma.

Inhibition of SNAIL function by *LINC00173* is not well understood. It is conceivable that *LINC00173* reduces the amount of SNAIL RNA by reducing SNAIL transcriptional efficiency and/or the stability of SNAIL mRNA. However, there was no significant change in the amount of SNAIL mRNA upon depletion of *LINC00173* (Figure S9). This suggests that *LINC00173* affects SNAIL protein and not *SNAIL* transcription and transcripts, which is consistent with the finding that *LINC00173* binds to SNAIL protein. Possible mechanisms by which *LINC00173* affects SNAIL protein include regulation of translation efficiency, post-translational modifications, stability control, and effects on SNAIL function. There are many possibilities for this, and here we suggest the following. If *LINC00173* binds to an E3 ligase other than SNAIL, it may reduce the stability of the SNAIL protein by promoting its proteasome degradation. Indeed, lncRNAs such as *LITATS1* [53], *OCC-1* [54], and *HOTAIR* [55] decrease the stability of target proteins. As a mechanism to inhibit the molecular function of SNAIL, if *LINC00173* masks the zinc finger motif that recognizes the E-box DNA binding sequence of SNAIL [19], it may affect the recruitment of SNAIL to its target region. An alternative mechanism to inhibit the molecular function of SNAIL is if *LINC00173* masks the SNAG domain, which is essential for the binding of transcriptional co-repressors, such as HDAC1/2, PRC2, LSD1/KDM1A, and SUV39H1, to SNAIL [20,21,22,23]. This may interfere with the binding of these transcriptional co-repressors to SNAIL. It is therefore important to clarify which region of SNAIL *LINC00173* binds to, what effect it has on SNAIL, and which transcriptional co-repressor(s) bind to SNAIL. We acknowledge this as a limitation of this study, and further detailed investigations are required.

In this study, we focused on the role of *LINC00173* in tumorigenesis and cancer progression. However, *LINC00173* plays a role in other diseases and processes, such as myasthenia gravis [56], hypertrophic scar [57], polycystic ovary syndrome [58], and regulation of cytokine production [59]. Indeed, among the genes whose expression is altered upon *LINC00173* depletion were some that have non-cancer functions and others whose functions are unknown. For example, IL15RA, whose expression decreased upon *LINC00173* depletion, is a neutrophil attractant [60], indicating that *LINC00173* may act as a tumor suppressor by promoting anticancer immunity. In addition, the RNA-seq data include a group of non-coding RNAs whose expression is altered upon *LINC00173* depletion, which may also have important functions. Therefore, the RNA-seq data we generated in this study will be a valuable resource for future analyses of *LINC00173* function.

## 4. Materials and Methods

### 4.1. Cell Culture

Dulbecco’s modified Eagle’s medium (DMEM) purchased from Sigma (Sigma-Aldrich, St. Louis, MO, USA, D5796), from FUJIFILM (FUJIFILM Wako Pure Chemical Corp., Osaka, Japan, 044-29765), and Leibovitz’s L-15 medium (Thermo Fisher Scientific, Waltham, MA, USA, 11415-064) were used for culturing A549, H1299, and MDA-MB-231 cells, respectively. Each culture medium was supplemented with 10% fetal bovine serum (FBS) purchased from Sigma (Sigma-Aldrich, 172012) for A549 or from Corning (Corning, Corning, NY, USA, 35-015-CV) for H1299 and MDA-MB-231, and 100 U/mL penicillin (Meiji Seika Pharma Co., Ltd., Tokyo, Japan, 01163) and 100 μg/mL streptomycin (Meiji Seika Pharma Co., Ltd., 02002) for each cell culture. All cells were maintained at 37 °C in an atmosphere containing 5% CO_2_. A549, H1299, and MDA-MB-231 cells were purchased from ATCC (Manassas, VA, USA). These catalog numbers are CCL-185, CRL-5083, and HTB-26, respectively.

### 4.2. Plasmids

DNA fragments, −1548/+52 of human *FHIT* and −1072/+67 of human *LINC00173* relative to the transcription initiation sites of each gene, were amplified by genomic DNA PCR and then cloned into luciferase reporter plasmid pGL4.10 (Promega, Madison, WI, USA, E6651) to generate p*FHIT*-Luc (−1548/+52) and p*LINC00173*-Luc (−1072/+67), respectively. Empty vector pcDNA3.1 was purchased from Invitrogen (Invitrogen, Carlsbad, CA, USA, V870-20). The plasmids, pCMV-β-galactosidase (β-gal) and pcDNA3-SNAIL-HA, were provided by Hidetoshi Hayashi (Nagoya City University, Nagoya, Japan) and Keiji Miyazawa (Yamanashi University, Chuo, Japan), respectively.

### 4.3. RNA Interference

For depletion of *LINC00173* and SNAIL, ASO and siRNA were used, respectively. ASO and siRNA were transfected into cells using Lipofectamine^TM^ RNAiMAX (Invitrogen, 13778-150) according to the manufacturer’s instructions. The *LINC00173* ASO, negative control ASO, and negative control siRNA were obtained from Qiagen (Qiagen, Hilden, Germany). *LINC00173* ASO: Antisense LNA^TM^ GapmeR Standard 339511, LG00803485-DDA (ASO-173#1) and LG00803490-DDA (ASO-173#2), negative control ASO: Antisense LNA^TM^ GapmeR control 339515, LG00000002-DDA (ASO-NC), and negative control siRNA: #1027310 (siCtrl), and SNAIL siRNA was obtained from Sigma-Aldrich (Sigma-Aldrich, 00039791 (siSNAIL). Target sequences of ASO and siRNA are listed in Supplementary Table S2.

### 4.4. Reverse Transcription Quantitative Polymerase Chain Reaction (RT-qPCR) Analysis

Total RNA was isolated from cells using an RNeasy Mini Kit (Qiagen, #74104), followed by reverse transcription using random hexanucleotide primers and reverse transcriptase SuperScript II (Invitrogen, #18064014). Quantitative PCR was carried out on a StepOnePlus system (Life Technologies, Carlsbad, CA, USA, 4376373) using SYBR^®^ Green Realtime PCR Master Mix (TOYOBO, Osaka, Japan, #QPS-201). Each expression value was normalized with *GAPDH*. Primer sequences are listed in Supplementary Table S3.

### 4.5. Immunoblot Analysis

Cells were lysed with lysis buffer [0.3% Triton X-100, 300 mM NaCl, 50 mM Tris-HCl, pH 7.5, protease inhibitor cocktail (Roche, Basel, Switzerland, 11697498001)] and then lysed cells were sonicated to prepare cell lysates using a Bioruptor sonicator (Cosmo Bio, Tokyo, Japan, UCS-250). Cell lysates were denatured in SDS sample buffer at 95 °C for 8 min. Then, the cell lysates were separated by SDS-PAGE and electrically transferred onto a polyvinylidene difluoride (PVDF) membrane (Millipore, Burlington, MA, USA, IPVH00010). Proteins blotted onto the membrane were visualized using primary antibodies and corresponding HRP-conjugated secondary antibodies and an enhanced chemiluminescence system (Bio-Rad, Hercules, CA, USA, 170-5061, or Cytiva, Tokyo, Japan, RPN2235). Each molecular weight of the bands is estimated using a protein marker, ExcelBand 3-color Pre-Stained Protein Ladder (SMOBIO Technology, Hsinchu, Taiwan, PM5100).

Antibodies used for immunoblotting were as follows: anti-SNAIL (Cell Signaling Technology, Danvers, MA, USA, #4719), anti-FHIT (Invitrogen, Waltham, MA, USA, #71-9000), anti-HA-peroxidase (Roche, 3F10), anti-β-actin (FUJIFILM Wako Pure Chemical Corp., 010-27841), horseradish peroxidase (HRP)-conjugated anti-rat IgG (Invitrogen, #65-9520), anti-rabbit IgG (Promega, Madison, WI, USA, W401B), and anti-mouse IgG (Promega, W402B).

### 4.6. RNA Sequencing Analyses

For the expression profiles of the A549 cells, total RNA was purified using an RNeasy Mini Kit (Qiagen) in accordance with the manufacturer’s instructions. The quality of RNA was evaluated by an Agilent 2200 TapeStation (Agilent Technologies, Santa Clara, CA, USA) and the quantity of RNA was measured using a NanoDrop spectrophotometer (Thermo Fisher Scientific, ND-1000). Preparation of the cDNA library and sequencing were commissioned to Macrogen Inc. (Seoul, Republic of Korea, https://macrogen.com). Alignment of sequence reads to the human reference genome (GRCh38.p13, release 102), calculation of TPM, and statistical analyses were performed with CLC genomics Workbench 20.0 (Qiagen). The RNA-seq data is deposited in DDBJ under accession number DRA017258.

For the identification of target genes for *LINC00173*, A549 cells were transfected with ASOs (ASO-NC, ASO-173#1, or ASO-173#2). At 24 h post-transfection, cells were harvested, and total RNA was extracted. Evaluation of the quality and quantity of RNA and RNA-seq analysis was performed as described above, except that a different human reference genome (GRCh38.p13, release 108), which was the latest version, was used. The RNA-seq data is deposited in DDBJ under accession number DRA017176.

### 4.7. RNA Immunoprecipitation Assay (RIP Assay)

RIP assays were executed as previously reported [61,62] with minor modifications. In brief, A549 cells were lysed with lysis buffer [0.3% Triton X-100, 300 mM NaCl, 50 mM Tris-HCl, pH 7.5, protease inhibitor cocktail (Roche), and RNase inhibitor (TOYOBO, SIN-201)] and then the lysed cells were sonicated to prepare cell lysates using a Bioruptor sonicator. The lysate (i.e., input sample) was incubated with an anti-SNAIL antibody (Cell Signaling Technology, #3879) or normal rabbit IgG (Cell Signaling Technology, #2729), which were pre-conjugated with Dynabeads^®^ protein G (Thermo Fisher Scientific, #10004D). After 2.5 h incubation, the beads were washed with washing buffer (50 mM Tris-HCl, pH 7.4, 150 mM NaCl, 1 mM MgCl_2_, 0.05% NP-40) using a magnetic stand for preparing immunoprecipitation (IP) samples. 10% of the IP samples and 1% of the input sample were subjected to immunoblot analysis (for details, please see Section 4.5, “*Immunoblot Analysis*”, as described above) to confirm the proper immunoprecipitaiton. From the remaining IP samples, co-precipitated RNA was purified using ISOGEN-LS (NIPPON GENE CO., LTD. Tokyo, Japan, 311-02621) in accordance with manufacturer’s instruction. Reverse transcription was performed using random hexanucleotide primers and reverse transcriptase SuperScript IV (Invitrogen, 18090050). The synthesized cDNA was subjected to quantitative PCR using a StepOnePlus system and SYBR^®^ Green Realtime PCR Master Mix to measure the RIP efficiency.

### 4.8. Luciferase Reporter Assay

Luciferase assays were executed as previously reported [62]. In brief, A549 cells were transfected with the reporter plasmid (p*FHIT*-Luc (−1548/+52) or p*LINC00173*-Luc (−1072/+67)), expression plasmid (pcDNA3-SNAIL-HA), empty vector (pcDNA3.1), and β-gal plasmid (pCMV-β-gal) using ViaFect reagent (Promega, E4981) in Opti-MEM (Invitrogen, 31985-070) in accordance with manufacturer’s instruction. At 48 h post-transfection, the cells were lysed with lysis buffer (Promega, E194A) and the luciferase activity was detected using Luciferase Assay System (Promega, E151A) with Luminescencer-JNR II (ATTO, Tokyo, Japan, AB-2300) and β-gal activity is detected using 2-Nitrophenyl β-D-Galactopyranoside (FUJIFILM Wako Pure Chemical Corp., 148-04693) as a substrate with Synergy H1 Multimode Microplate Reader (Agilent Technologies). Luciferase activity was normalized to β-gal activity in the same sample.

### 4.9. In Silico Analysis

Expression datasets for *LINC00173*, *FHIT*, and *SNAIL*, and survival information for lung adenocarcinoma, breast invasive carcinoma, lung squamous cell carcinoma, acute myeloid leukemia, and cervical squamous cell carcinoma were obtained from cBioPortal (https://www.cbioportal.org/). These original data are deposited in the PanCancer Atlas of The Cancer Genome Atlas (TCGA). Samples missing either expression data or survival status were excluded from the analysis. For prognostic analysis, the gene expression dataset was divided into two groups (low and high) based on cutoff values determined receiver operating characteristic (ROC) curve analyses. Survival curves were developed based on the above cutoff values. For the correlation between each gene, the expression data was analyzed in two groups based on median values. These in silico analyses were performed using EZR software version 1.41 (Saitama Medical Center, Jichi Medical University, Saitama, Japan).

### 4.10. Statistical Analysis

Student’s t-test and one-way ANOVA with the Holm–Bonferroni method were used to compare two- and multi-group data in the experiment, respectively. The cutoff values for the expression levels of *LINC00173*, *FHIT*, and *SNAIL* were determined by ROC curve analyses using the Youden index [maximum value of (sensitivity) + (specificity-1)]. Overall survival was evaluated by means of the Kaplan–Meier method, and survival curves were compared using the log-rank test. Hazard ratio was calculated by Cox proportional hazards analysis. Correlation between the expression of each gene was evaluated by Peason’s χ^2^ test. Statistical analyses were carried out using EZR software version 1.41. Values of *p* < 0.05 were considered statistically significant in all analyses.

## Data Availability

The RNA-seq data in this study have been deposited in the Sequence Read Archive (SRA) of the DNA Data Bank of Japan (DDBJ; https://www.ddbj.nig.ac.jp/index-e.html, accessed on 8 November 2023) under accession numbers DRA017176 and DRA017258.

## Supplementary Materials

The following supporting information can be downloaded at: https://www.mdpi.com/article/10.3390/ijms242317011/s1. Reference [63] was cited in Supplementary Materials.
