# Supplementary material for: Expression of Tumor Suppressor FHIT Is Regulated by the LINC00173-SNAIL Axis in Human Lung Adenocarcinoma
Source: Int J Mol Sci. 2023 Nov 30;24(23):17011. doi: 10.3390/ijms242317011 (PMC10707390; doi:10.3390/ijms242317011)
Supplement: Supplementary file 1 [file ijms-24-17011-s001.zip › ijms-2736890-supplementary.pdf]

Supplementary information

Expression of Tumor Suppressor *FHIT* Is Regulated by the *LINC00173*-SNAIL Axis in Human Lung Adenocarcinoma

Takahito Suzuki[1, 2, †], Satoshi Sakai[1, †, *], Kosuke Ota[1], Mika Yoshida[1], Chiharu Uchida[3], Hiroyuki Niida[1], Takafumi Suda[2], Masatoshi Kitagawa[1] and Tatsuya Ohhata[1, *]

[1] Department of Molecular Biology, Hamamatsu University School of Medicine, Hamamatsu 431-3192, Japan

[2] Second Division, Department of Internal Medicine, Hamamatsu University School of Medicine, Hamamatsu 431-3192, Japan

[3] Advanced Research Facilities & Services, Preeminent Medical Photonics Education & Research Center, Hamamatsu University School of Medicine, Hamamatsu 431-3192, Japan

* Correspondence: ohhata@hama-med.ac.jp (T.O.); ssakai@hama-med.ac.jp (S.S.)

[†] These authors contributed equally to this work.

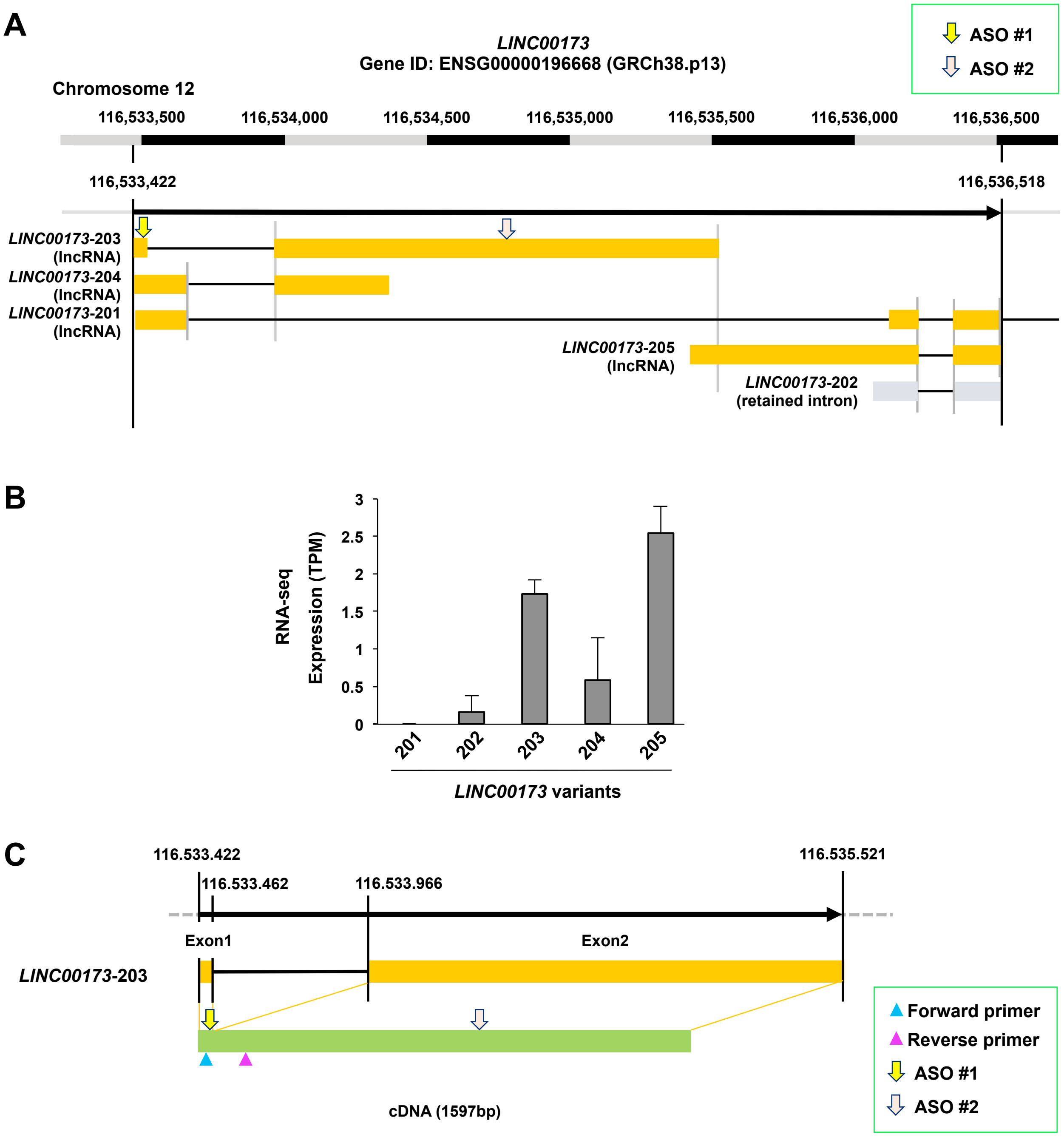

Figure S1. Gene map of *LINC00173*. (**A**) Gene map of five *LINC00173* variants from the Ensembl genome browser (GRCh38.p13). (**B**) Expression profiles of five *LINC00173* variants in A549 cells from RNA-seq data. (**C**) Gene map of *LINC00173*-203, focused on in this study. Arrows and arrowheads indicate target sites of antisense oligonucleotides (ASOs) and PCR primers, respectively. ASO #1 and #2 target the spliced form and exon 2 of *LINC00173*-203, respectively, and specifically recognize *LINC00173*-203 but not other variants. The primer set also only recognizes the spliced variant *LINC00173*-203, but not others.

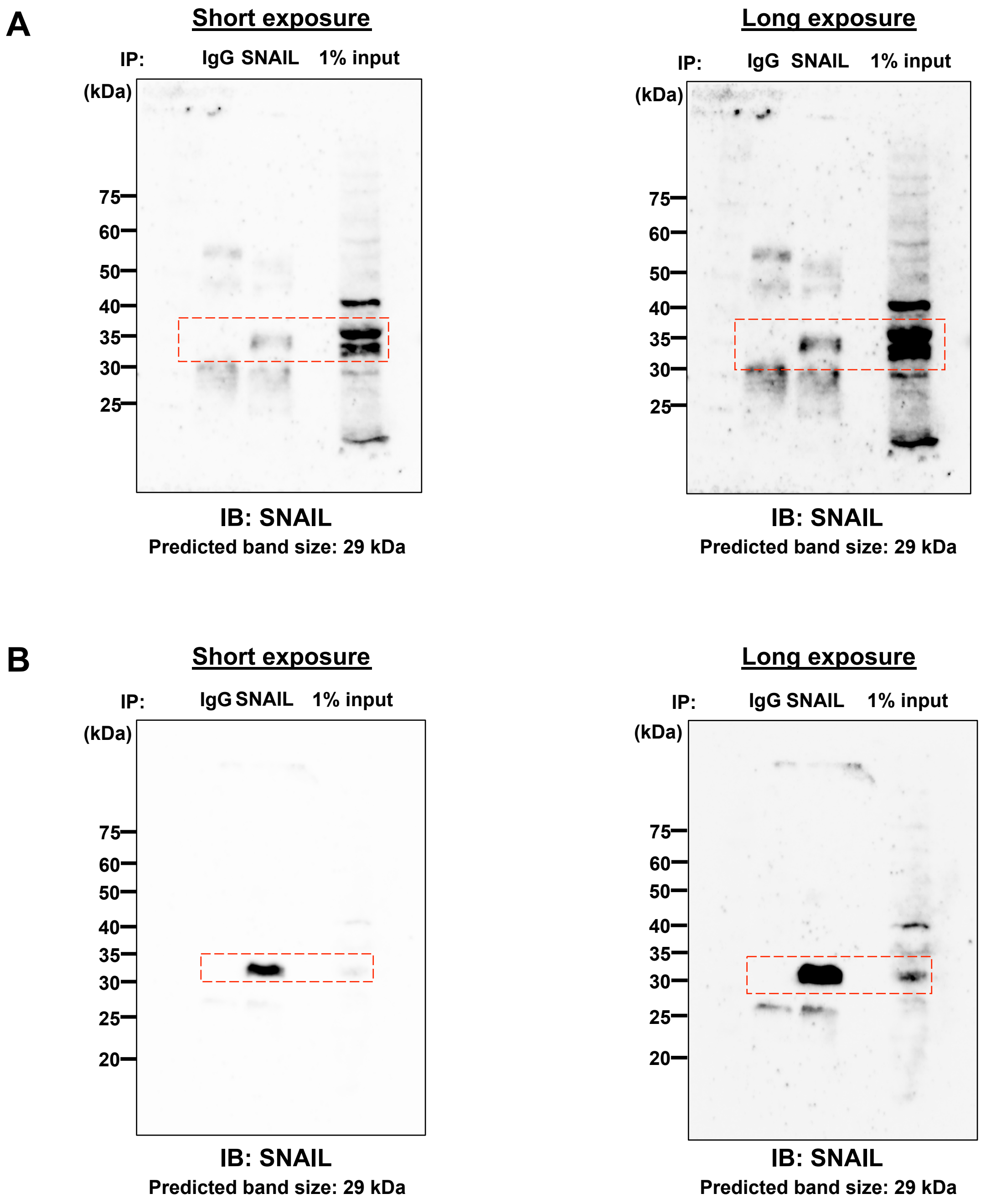

Figure S2. Uncropped images of IP-IB, related to Figure 1. (**A,B**) Results of RIP-IB using an anti-SNAIL antibody in the 1st experiment (**A**) and 2nd experiment (**B**), detected with short exposure (left) and long exposure (right). The area enclosed by the red dotted line is shown at the bottom of Figure 1B.

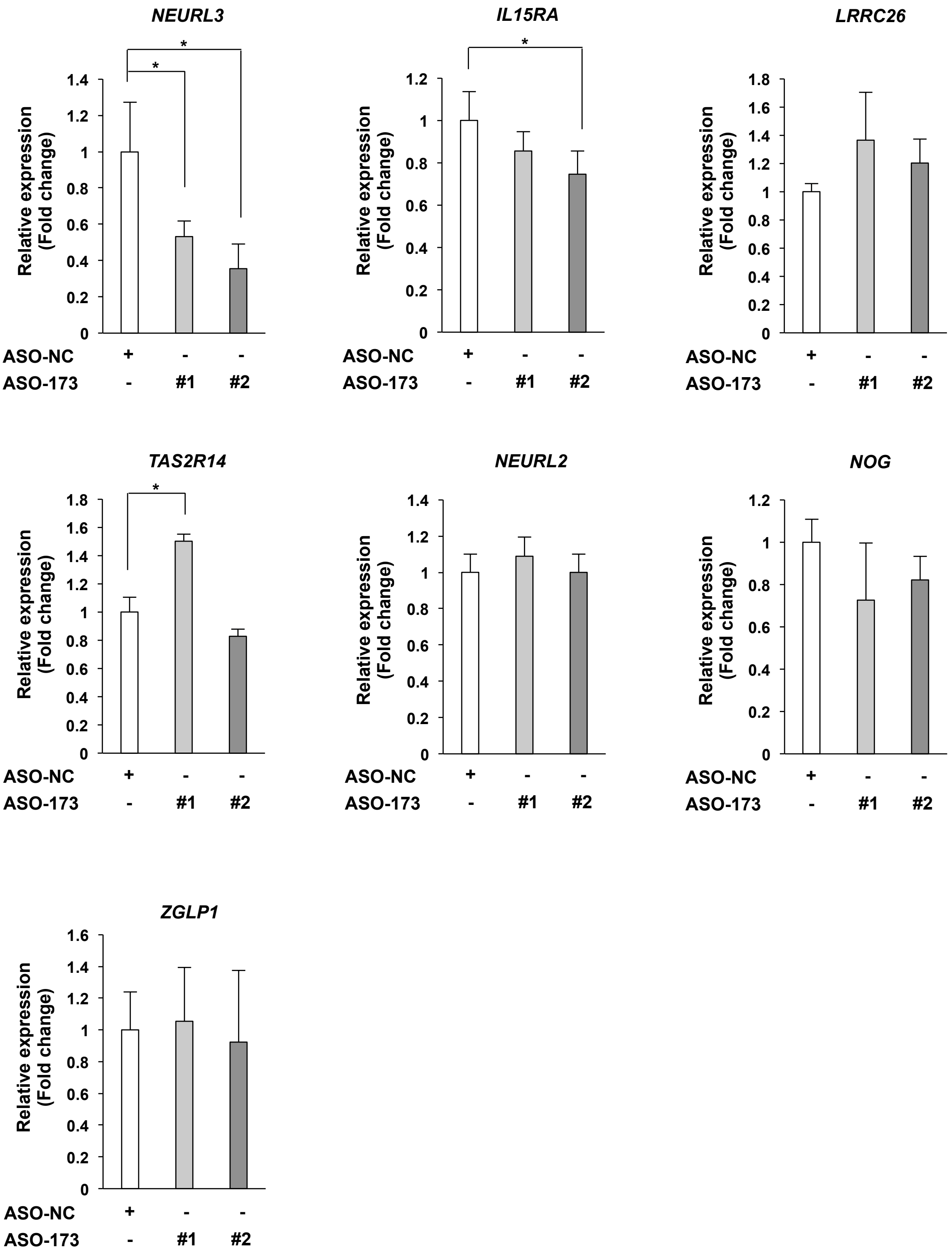

Figure S3. Expression analysis of other candidate target genes of *LINC00173*. RT-qPCR determination of the relative expression of seven genes, *NEURL3*, *IL15RA*, *LRRC26*, *TAS2R14*, *NEURL2*, *NOG*, and *ZGLP1*, which are candidate target genes of *LINC00173*, upon depletion of *LINC00173*. Three biological replicates, normalized to *GAPDH*, and the mean ± SD relative to the mean of each ASO-NC is shown. *: $p < 0.05$, relative to each ASO-NC transfection, Student's t-test.

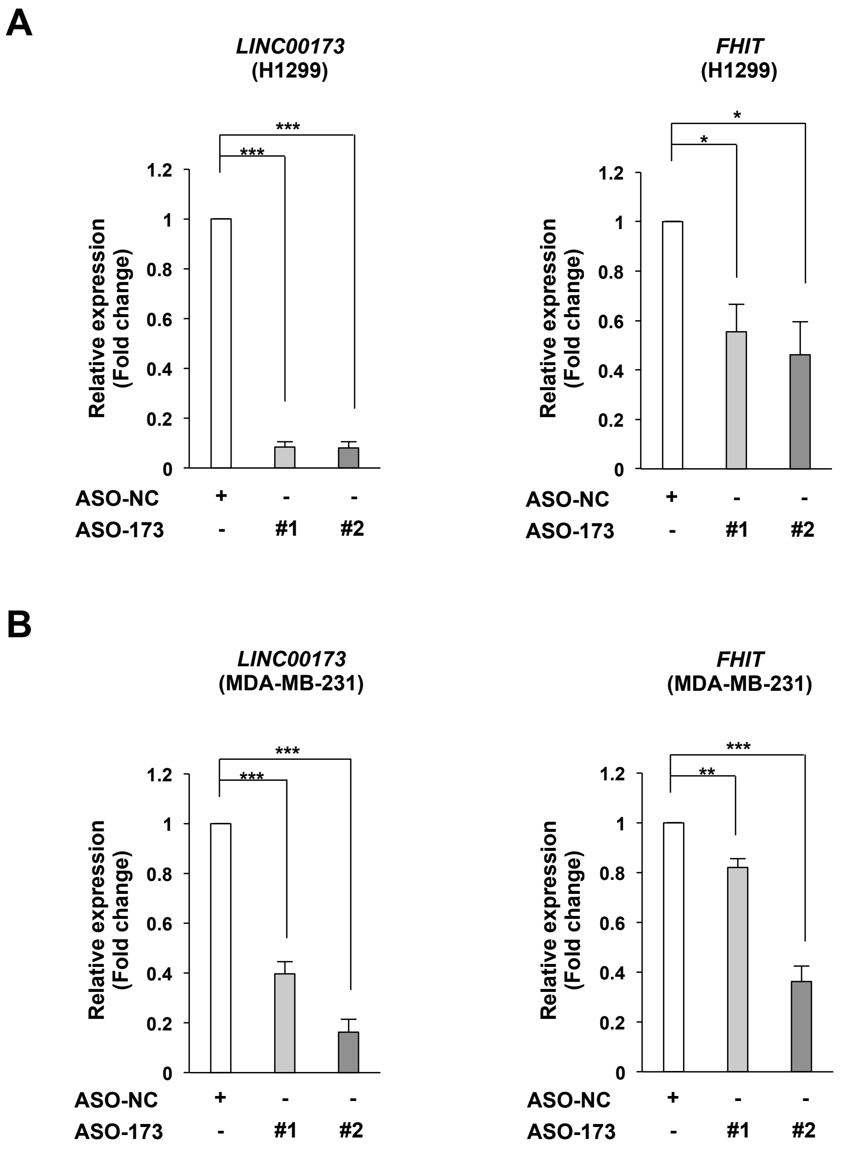

Figure S4. Expression of *LINC00173* and *FHIT* in several cell lines. (**A,B**) RT-qPCR determination of the relative expression of *LINC00173* and *FHIT* in H1299 cells (**A**) and MDA-MB-231 cells (**B**). Three biological replicates, normalized to *GAPDH*, and the mean ± SD relative to the mean of each ASO-NC are shown. *: $p < 0.05$, **: $p < 0.01$, and ***: $p < 0.001$, relative to each ASO-NC transfection, Student's t-test.

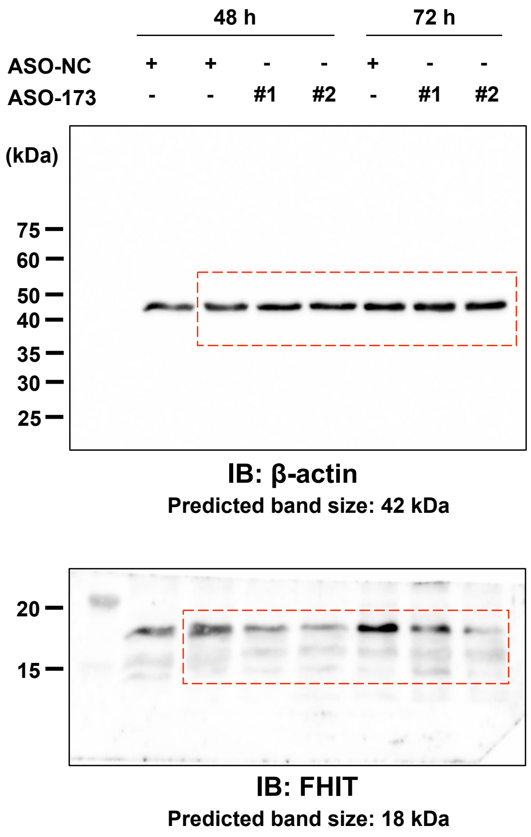

Figure S5. Uncropped images of western blotting, related to Figure 2. Uncropped images of western blotting from Figure 2E and the approximate cropped regions for Figure 2E indicated by dotted-red squares are shown.

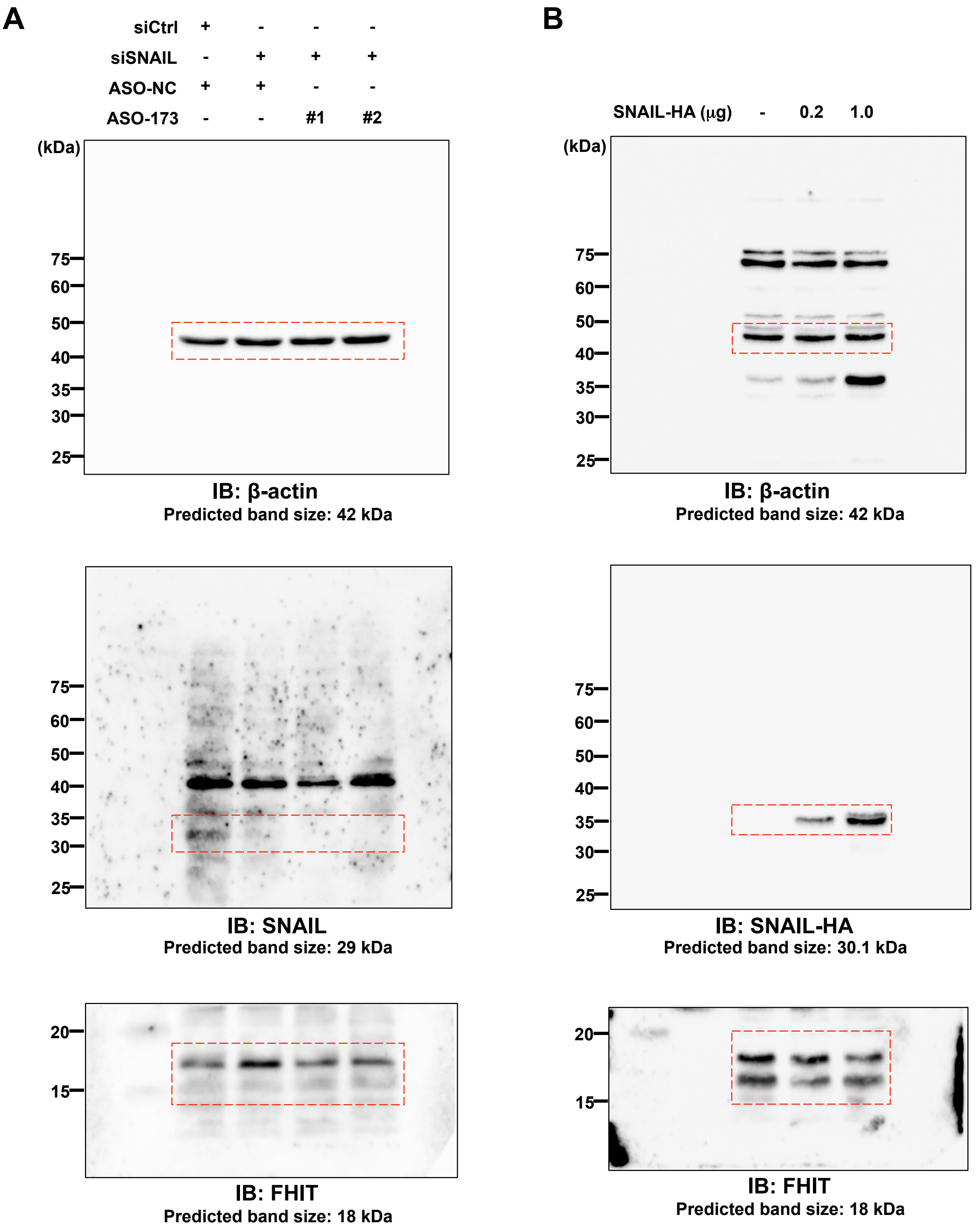

Figure S6. Uncropped images of western blotting, related to Figure 3. (**A**,**B**) Uncropped images of western blotting from Figure 3D (**A**) and 3E (**B**) and the approximate cropped regions for Figure 3D (**A**) and 3E (**B**) indicated by dotted-red squares are shown.

A **Breast cancer**

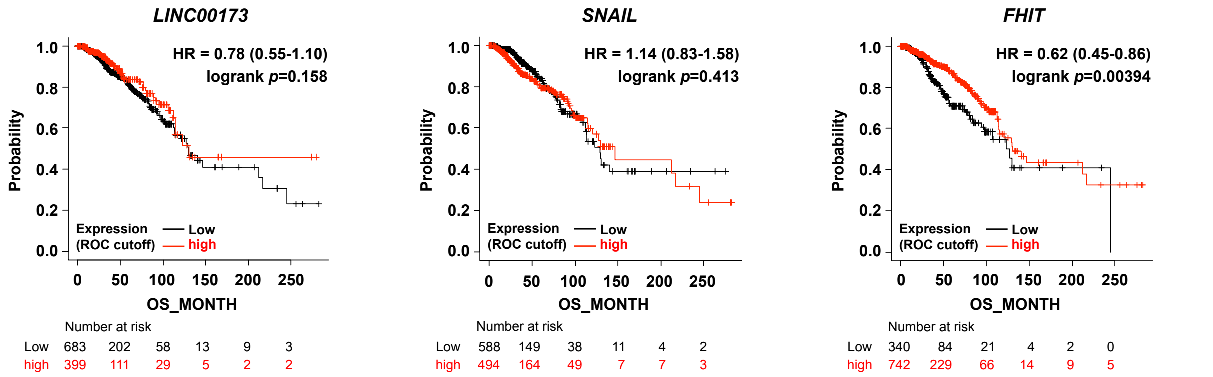

B **Breast cancer**

		SNAIL (n=1082) Low	*SNAIL* (n=1082) High	*p* value
LINC00173 (n=1082)	Low	214	327	9.77×10^{-12}
	High	327	214	

		FHIT (n=1082) Low	*FHIT* (n=1082) High	*p* value
SNAIL (n=1082)	Low	227	314	1.70×10^{-7}
	High	314	227	

		FHIT (n=1082) Low	*FHIT* (n=1082) High	*p* value
LINC00173 (n=1082)	Low	353	188	2.03×10^{-23}
	High	188	353	

C **Lung squamous cell carcinoma**

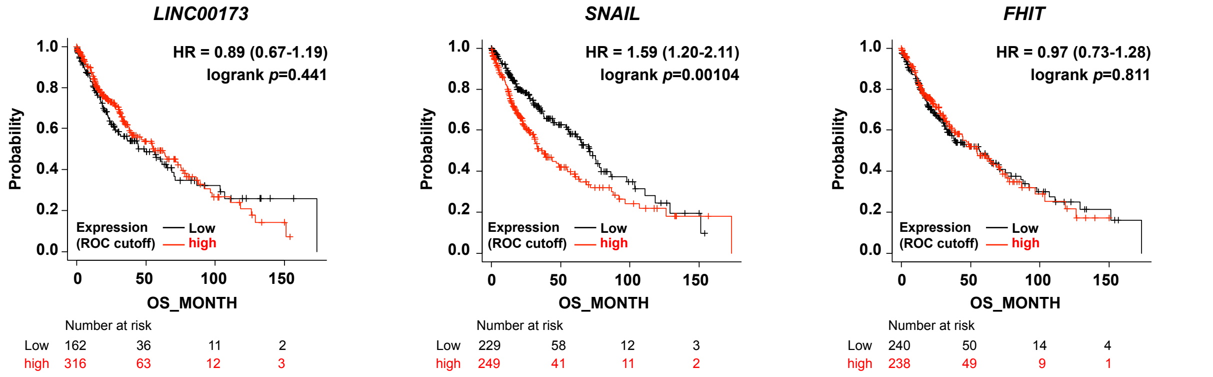

D **Lung squamous cell carcinoma**

		SNAIL (n=478) Low	*SNAIL* (n=478) High	*p* value
LINC00173 (n=478)	Low	102	137	0.00187
	High	137	102	

		FHIT (n=478) Low	*FHIT* (n=478) High	*p* value
SNAIL (n=478)	Low	125	114	0.360
	High	114	125	

		FHIT (n=478) Low	*FHIT* (n=478) High	*p* value
LINC00173 (n=478)	Low	134	105	0.0104
	High	105	134	

Figure S7. Impact of the *LINC00173*-SNAIL-FHIT axis on human breast cancer and lung squamous cell carcinoma. (**A,C**) Prognostic analysis of patients with breast cancer (**A**) and lung squamous cell carcinoma (**C**) expressing *LINC00173*, *SNAIL*, and *FHIT* using Kaplan-Meier analysis. (**B,D**) Correlation analysis between *LINC00173* and *SNAIL*, *SNAIL* and *FHIT*, and *LINC00173* and *FHIT* in human breast cancer (**B**) and lung squamous cell carcinoma (**D**) using the χ^2 test.

A

Acute myeloid leukemia

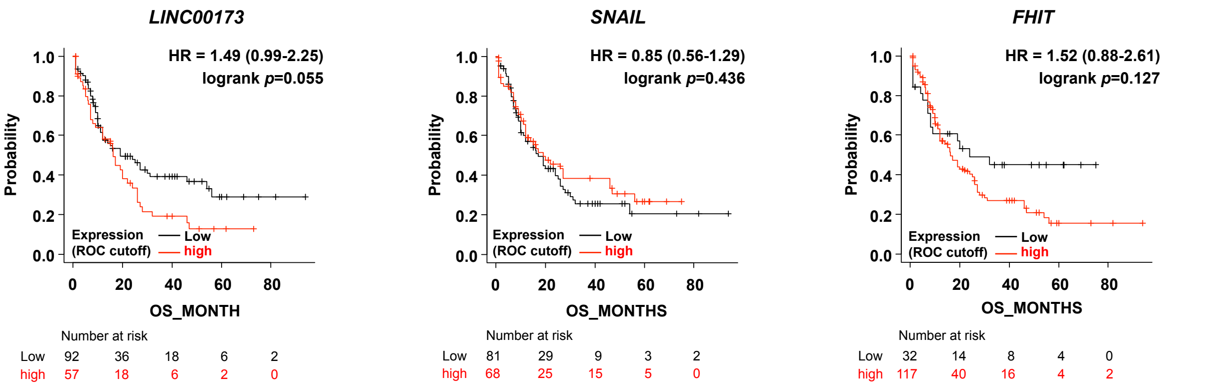

B

Acute myeloid leukemia

		SNAIL (n=149) Low	*SNAIL* (n=149) High	*p* value
LINC00173 (n=149)	Low	48	27	0.0014
	High	27	47	

		FHIT (n=149) Low	*FHIT* (n=149) High	*p* value
SNAIL (n=149)	Low	33	42	0.164
	High	42	32	

		FHIT (n=149) Low	*FHIT* (n=149) High	*p* value
LINC00173 (n=149)	Low	35	40	0.461
	High	40	34	

C

Cervical squamous cell carcinoma

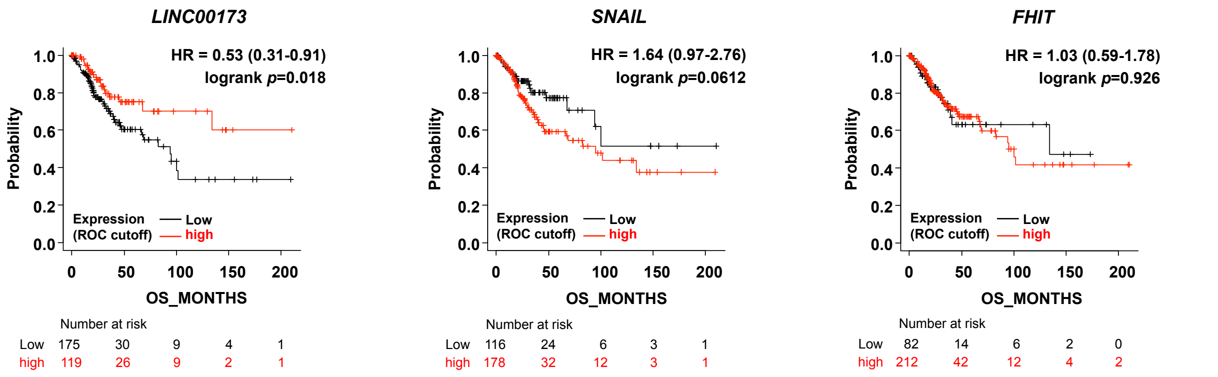

D

Cervical squamous cell carcinoma

		SNAIL (n=294) Low	*SNAIL* (n=294) High	*p* value
LINC00173 (n=294)	Low	69	78	0.351
	High	78	69	

		FHIT (n=294) Low	*FHIT* (n=294) High	*p* value
SNAIL (n=294)	Low	76	71	0.641
	High	71	76	

		FHIT (n=294) Low	*FHIT* (n=294) High	*p* value
LINC00173 (n=294)	Low	72	75	0.816
	High	75	72	

Figure S8. Impact of the *LINC00173*-SNAIL-FHIT axis on human acute myeloid leukemia and cervical squamous cell carcinoma. (**A,C**) Prognostic analysis of patients with acute myeloid leukemia (**A**) and cervical squamos cell carcinoma (**C**) expressing *LINC00173*, *SNAIL*, and *FHIT* using Kaplan-Meier analysis. (**B,D**) Correlation analysis between *LINC00173* and *SNAIL*, *SNAIL* and *FHIT*, and *LINC00173* and *FHIT* in human acute myeloid leukemia (**B**) and cervical squamous cell carcinoma (**D**) using the χ^2 test.

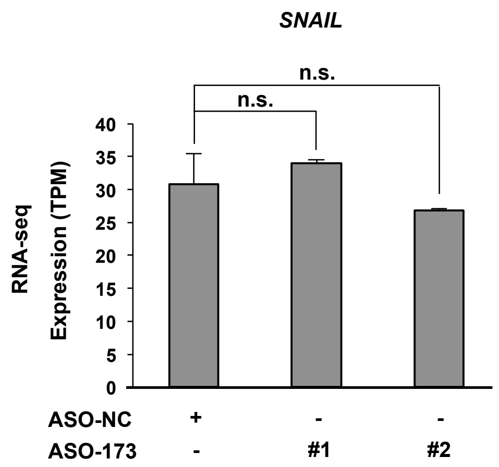

Figure S9. Expression analysis of *SNAIL* upon depletion of *LINC00173*. The RNA-seq data representing the expression of *SNAIL* is shown. Two biological replicates, mean ± SD, n.s.: not significant, Student's t-test.

Table S1. List of candidate genes for the *LINC00173* target in A549 cells

	Gene Symbol	Gene ID	Baseline TPM	Fold change by ASO #1	*p*-value Baseline vs. #1	Fold change by ASO #2	*p*-value Baseline vs. #2
**1**	NEURL3	ENSG163121	2.335	0.474	0.247596918	0.365	0.231302513
**2**	IL15RA	ENSG134470	2.328	0.439	0.030033822	0.459	0.124004326
**3**	LRRC26	ENSG184709	1.831	0.474	0.233738972	0.273	0.175470230
**4**	TAS2R14	ENSG212127	1.454	0.465	0.224119823	0.366	0.187257446
**5**	**FHIT**	ENSG189283	1.277	0.399	0.024171634	0.170	0.016859634
**6**	NEURL2	ENSG124257	1.195	0.000	0.262262838	0.197	0.344389932
**7**	NOG	ENSG183691	1.133	0.484	0.095465966	0.190	0.037749551
**8**	ZGLP1	ENSG220201	1.001	0.423	0.071720878	0.299	0.059279132

Table S2. Information for ASO and siRNA sequences

Antisense oligo nucleotide sequence

Oligo name	Target sequence
ASO-173#1	TCAAGCGCTTAGAGCA
ASO-173#2	GCACGCCCACTTTTTA
Negative control	AACACGTCTATACGC

siRNA sequence

Oligo name	Target sequence
SNAIL	CCAUGGAAUUCCCUCCUGA
Negative control	TTCTCCGAACGTGTCACGT

Table S3. Information for primer sequences

PCR products	Primer sequence	Intron	Expected length	Application	References
LINC00173		Yes	113 bp	RIP-qPCR (Fig 1C), RT-qPCR (Figs 2C, 2D, 3C, 3D, S4A, S4B)	
LINC00173-F1	AGGCTCCCACCTGCTCTAAG				
LINC00173-R1	CAGGACTTAGCTTTGCTCTTGC				
HDAC4-AS1		No	88 bp	RIP-qPCR (Fig 1C)	
HDAC4-AS1-F1	GCTTCGATTGTAGGCCATTC				
HDAC4-AS1-R1	AGTTTCCCCCACCAAGAATC				
LINC01816		No	121bp	RIP-qPCR (Fig 1C)	
LINC01816-F1	CGTGCTTCCTCCTAACCACT				
LINC01816-R1	ATGAAACAAGGTCGGGAAGG				
LINC02535		No	169 bp	RIP-qPCR (Fig 1C)	
LINC02535-F1	CAAGGCTGGAGGATAGCTTG				
LINC02535-R1	AGCCTCCCACACTAAAGCAA				
OGFRP1		No	108 bp	RIP-qPCR (Fig 1C)	
OGFRP1-F1	GGAGGGCCTTAAAACAGAGG				
OGFRP1-R1	TTCATCTTTTTCCCCTGCAC				
FHIT		Yes	149 bp	RT-qPCR (Figs 2C, 2D, 3C, 3D, S4A, S4B)	
FHIT-F1	AAGTGGCCGATTTGTTTCAG				
FHIT-R1	CTTCCTGGGAAGAACATGGA				
NEURL3		No	91 bp	RT-qPCR (Fig S3)	
NEURL3-F1	TTCCCCAACAGGATAGCAAG				
NEURL3-R1	GCCACCCCATCTCTAAACAA				
IL15RA		No	91 bp	RT-qPCR (Fig S3)	
IL15RA-F1	GCTGATGCCTTCAAAATCACC				
IL15RA-R1	CTTGGCTGTTGTCTGAGAGG				
LRRC26		No	66 bp	RT-qPCR (Fig S3)	
LRRC26-F1	AGCTTCCCCCATGCCTTT				
LRRC26-R1	TCGGTCTGTGTCGCTTGTT				
TAS2R14		No	122 bp	RT-qPCR (Fig S3)	
TAS2R14-F1	GGTGTGTGTCTGTGTTTTTCCC				
TAS2R14-R1	AAAAGTACCGAGGCCTGTAGC				
NEURL2		No	161 bp	RT-qPCR (Fig S3)	
NEURL2-F1	CCACCTGCCCAAAGAACTTA				
NEURL2-R1	TGTCAGCATCGGCTGTTTAT				
NOG		No	148 bp	RT-qPCR (Fig S3)	
NOG-F1	TTTATATTCCAGTGCCCTTCG				
NOG-R1	GAGGTCCAAGGAAAACCAAA				
ZGLP1		No	73 bp	RT-qPCR (Fig S3)	
ZGLP1-F1	GGCGCTGTGCTTCCTGTC				
ZGLP1-R1	TTGCAGAGAGGGGTCCCATC				
GAPDH		Yes	87 bp	RT-qPCR (Figs 2C, 2D, 3C, 3D, S3, S4A, S4B)	Sakai et al, 2019
GAPDH-F1	TGCACCACCAACTGCTTAGC				
GAPDH-R1	GGCATGGACTGTGGTCATGAG				
SNAIL		No	100 bp	RT-qPCR (Figs 3C, 3D)	Kawata et al, 2012
Snail-F1	TTCTCACTGCCATGGAATTCC				
Snail-R1	GCAGAGGACACAGAACCAGAAA				

References

1. Kawata, M.; Koinuma, D.; Ogami, T.; Umezawa, K.; Iwata, C.; Watabe, T.; Miyazono, K. TGF-β-induced epithelial-mesenchymal transition of A549 lung adenocarcinoma cells is enhanced by pro-inflammatory cytokines derived from RAW 264.7 macrophage cells. *J Biochem* **2012**, *151*, 205–216.

2. Sakai, S.; Ohhata, T.; Kitagawa, K.; Uchida, C.; Aoshima, T.; Niida, H.; Suzuki, T.; Inoue, Y.; Miyazawa, K.; Kitagawa, M. Long Noncoding RNA ELIT-1 Acts as a Smad3 Cofactor to Facilitate TGFβ/Smad Signaling and Promote Epithelial-Mesenchymal Transition. *Cancer Res.* **2019**, *79*, 2821–2838.
